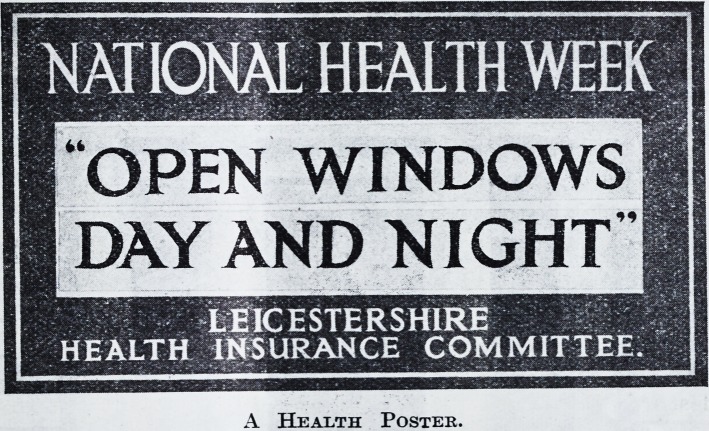# The Health Week Poster

**Published:** 1924-10

**Authors:** 


A Health Poster.

### THE HEALTH WEEK POSTER.

In the issue of posters for Health Week simplicity and boldness are the main considerations to be borne in mind. Too much subject-matter is fatal for publicity purposes—it merely confuses the eye. Small, distinctive posters, such as the one in use at Leicester, which we reproduce on this page, may be displayed in all manner of places—factories, warehouses, shops and surgeries. Trams and motor vehicles are also ideal positions in which to show health posters. The Leicestershire Health Insurance Committee is distributing 40,000 little "Health Grams" among the children for the forthcoming Health Week from October 5 to 11. They are ingenious, often amusing, and well calculated to remain in a child's memory. Number 1 is the "Song of the Fly" which takes a season ticket from the rubbish heap to the milk jug:—

Straight from the rubbish heap I come,
I never wash my feet,
And every single chance I get,
I walk on what you eat.

## Figures and Tables

**Figure f1:**